# Supplementary material for: Stacking Model‐Based Classifiers for Dealing With Multiple Sets of Noisy Labels
Source: Biom J. 2025 Mar 12;67(2):e70042. doi: 10.1002/bimj.70042 (PMC11898607; doi:10.1002/bimj.70042)
Supplement: Supplementary file 1 — Supporting Information [file BIMJ-67-e70042-s001.zip › density-based-ensemble-model-main/competitors_real_data/netlab-master/netlab-master/help/mlpprior.htm]

Netlab Reference Manual mlpprior


# mlpprior

## Purpose

Create Gaussian prior for mlp.

## Synopsis

```
prior = mlpprior(nin, nhidden, nout, aw1, ab1, aw2, ab2)
```

## Description

`prior = mlpprior(nin, nhidden, nout, aw1, ab1, aw2, ab2)`
generates a data structure
`prior`, with fields `prior.alpha` and `prior.index`, which
specifies a Gaussian prior distribution for the network weights in a
two-layer feedforward network. Two different cases are possible. In
the first case, `aw1`, `ab1`, `aw2` and `ab2` are all
scalars and represent the regularization coefficients for four groups
of parameters in the network corresponding to first-layer weights,
first-layer biases, second-layer weights, and second-layer biases
respectively. Then `prior.alpha` represents a column vector of
length 4 containing the parameters, and `prior.index` is a matrix
specifying which weights belong in each group. Each column has one
element for each weight in the matrix, using the standard ordering as
defined in `mlppak`, and each element is 1 or 0 according to
whether the weight is a member of the corresponding group or not. In
the second case the parameter `aw1` is a vector of length equal to
the number of inputs in the network, and the corresponding matrix
`prior.index` now partitions the first-layer weights into groups
corresponding to the weights fanning out of each input unit. This
prior is appropriate for the technique of automatic relevance
determination.

## See Also

`mlp`, `mlperr`, `mlpgrad`, `evidence`

---

**Pages:**
Index


---

Copyright (c) Ian T Nabney (1996-9)
